# Nutritional Assessment of Greek Liver Cirrhosis Patients: Mini Nutritional Assessment Predicts Mortality

**DOI:** 10.3390/healthcare10050859

**Published:** 2022-05-06

**Authors:** Mairi Koulentaki, Ioannis Drygiannakis, Aikaterini Mantaka, Evangelos Moschapidakis, Anna Chalkiadaki, Aikaterini Augoustaki, Aspasia Spyridaki, Elias Kouroumalis, Anastasia Markaki

**Affiliations:** 1 Department of Gastroenterology, University Hospital of Heraklion, 71500 Heraklion, Greece; mkoulentaki@yahoo.gr (M.K.); idrygiannakis@gmail.com (I.D.); katmant@gmail.com (A.M.); augoustaki@gmail.com (A.A.); kouroumi@uoc.gr (E.K.); 2 Department of Nutrition and Dietetics Sciences, Hellenic Mediterranean University, 72300 Sitia, Greece; e.moschapidakis@gmail.com (E.M.); annachalkiadakh@gmail.com (A.C.); aspaspyridaki@hmu.gr (A.S.)

**Keywords:** nutritional status, bioelectric impedance, model for end-stage liver disease, anthropometrics, handgrip strength

## Abstract

Malnutrition is highly prevalent in liver cirrhosis (LC). It increases as the severity of the disease progresses and it is related to poor survival. The objectives of the study were the nutritional assessment of Greek LC patients, using various nutritional assessment and screening tools, and the comparison of their predictive value for mortality. In total, 137 (77 male) consecutive LC patients (median age: 67 years) were assessed with subjective global assessment (SGA) and mini nutritional assessment (MNA) questionnaires, anthropometrics, handgrip strength (HGS) tests, and bioelectric impedance analysis (BIA), in comparison to a control group of 148 healthy people. Disease severity was assessed using the model for end-stage liver disease (MELD) scores. Patients were followed up for a median of 19 months. Survival curves were calculated using the Kaplan–Meier method. In total, 60% and 43% of patients were of adequate nutritional status by SGA and MNA, respectively, which was confirmed by most anthropometric measurements. MNA and SGA scores correlated significantly with anthropometrics and BIA-derived parameters. Besides the MELD score, mid-arm circumference (MAC), triceps skinfold (TSF), BIA’s phase angle (Pha), and MNA predicted mortality in cirrhotic patients. The nutritional assessment demonstrated an unexpectedly high prevalence of well-nourished LC patients. MNA was a strong predictor of mortality.

## 1. Introduction

Malnutrition is frequent in liver cirrhosis (LC) and increases with disease severity, as assessed by Child–Turcotte–Pugh (CTP) and end-stage liver disease (MELD) scores. Its prevalence is 46% and 95% in CTP stage A and C, respectively, and it is associated with increased morbidity and mortality, irrespective of LC stage [1].

There are no gold-standard methods to assess nutritional status in LC due to ascites, edema, or obesity. Τhe latest European Society for Parenteral and Enteral Nutrition (ESPEN) guidelines recommend using the subjective global assessment (SGA) as a screening tool, as well as methods such as anthropometrics, handgrip strength (HGS), and bioelectric impedance analysis (BIA) for parameter Phi angles (Pha), as part of a detailed assessment [2]. Although the validity of BIA in LC has been disputed due to the erroneous estimation of body fluid compartment, Pha correlates with liver disease severity and is not affected by the hydration status [3]. A cut-off value of 5.44° has been proposed for malnutrition in LC, while values ≤4.9° have been associated with increased mortality [4]. Moreover, BIA-derived body cell mass (BCM) as well as intracellular and extracellular water (ICW and ECW) have been shown to be reliable with or without ascites [5].

SGA has been widely used in cirrhotic patients [6]. Although it may predict severity and short-term survival in these patients, it has been found to underestimate the severity of malnutrition [7]. Meanwhile, the mini-nutritional assessment (MNA) is a validated instrument initially designed to identify nutritional status in the elderly population, which has gained worldwide acceptance [8]. Research has shown that it has strong sensitivity, specificity, and predictive value for malnutrition [9]. MNA is also being used in non-geriatric patient populations (e.g., oncological, end-stage renal disease, and heart failure patients) and has recently been suggested as an effective nutritional screening tool in LC patients [10,11,12,13].

Given that there are limited data on the nutritional status of Greek LC patients [14], the objectives of the study were: (a) to assess the nutritional status of Greek LC patients in Crete, using various assessment tools, including SGA, MNA, HGS, BIA, and anthropometry; and (b) to define the method that best correlates with mortality, using the MELD score as the gold standard.

## 2. Materials and Methods

### 2.1. Patients’ Recruitment

This was a single-center prospective study. LC diagnosis was based on liver biopsy (all patients with compensated cirrhosis) and/or clinical evidence of decompensation combined with endoscopic and radiological findings. Follow-up data were retrieved from clinical records and/or death certificates by personnel blind to anthropometric, body composition, and laboratory assessments. Patients were followed up for all-cause mortality from November 2013 to December 2016. Inclusion criteria were: (1) LC, (2) age ≥ 18 years, (3) an ability to communicate effectively, and (4) abstinence from alcohol for at least 1 year. Exclusion criteria were malignant disease, concurrent inflammatory illness, hepatic encephalopathy, heart failure, renal failure, sepsis, and an unwillingness to participate.

The study enrolled 137 cirrhotics (68 compensated, 69 decompensated) patients, as well as a control group of 148 healthy people without significant differences regarding sex and age, from the family environment. Written informed consent was obtained, and the study was conducted in accordance with the 1975 Declaration of Helsinki (6th revision, 2008). The protocol was approved by the Ethics Committee of the University Hospital of Heraklion, Greece (approval number 11886, 18 October 2013), and was conducted in the hospital.

### 2.2. Anthropometric Evaluation

Body weight and height were assessed. Body mass index (BMI) was calculated as body weight in kilograms divided by height in meters squared (kg/m^2^). Ascites was controlled with the use of diuretics. A BMI cut-off value of <22 kg/m^2^ was used for the diagnosis of malnutrition, which has been suggested for LC patients without ascites [15].

Triceps skinfold (TSF) and mid-arm circumference (MAC) were measured on the right side with the arm relaxed. Mid-arm muscle circumference (MAMC) and the arm muscle area (AMA) were calculated as follows: MAMC(cm) = MAC(cm) − 3.14 × TSF(cm) and AMA(cm) = [MAC(cm) − 3.14 × TSF(cm)]^2^/4π, respectively [16]. A digital scale (Seca 703, Hamburg, Germany), a stadiometer (Seca 220, Hambourg, Germany), a set of Harpenden skinfold calipers (HSB-BI, British Indicators, West Sussex, England), and inextensible tape were used. The averages of three measurements were recorded. All measurements were performed by a single trained dietitian.

### 2.3. Handgrip Strength

A mechanical handgrip dynamometer with an adjustable handle (Saehan Smedley type, spring dynamometer, New York, NY, USA) was used with subjects in a sitting position using the non-dominant hand at a 90° angle. The mean of three measurements of maximal effort with 30 s intervals was recorded. The suggested EWGSOP cut-off values for reduced handgrip strength (HGS) were used (males < 27 kg, females < 16 kg) [17].

### 2.4. Body Composition Analysi

A monofrequency BIA analyzer with a 50 kHz single-frequency system and tetrapolar electrodes was used (BIA-101, RJL/Akern Systems, Clinton Township, MI, USA). Impedance measurements were taken with subjects lying supine with their arms relaxed away from the trunk and with their thighs separated. Two sets of electrodes were attached on the dominant side of the body: the first on the dorsum of the hand and on the wrist joint, and the second on the dorsal surface of the foot and the ankle joint. The average of the two readings was recorded. Subsequently, Pha, total body water (TBW), extracellular water (ECW), intracellular water (ICW), body cell mass (BCM), fat mass (FM), fat free mass (FFM), and muscle mass (MM) were calculated [5,16,18]. From the latter metric, skeletal muscle index (SMI) was calculated [19]. The cut-offs used for SMI were <7.0 kg/m^2^ for men and <5.5 kg/m^2^ fοr women, according to the updated criteria developed by the European Working Group on Sarcopenia in Older People (EWGSOP-2, 2019) [17].

### 2.5. Disease Severity

The severity of liver disease was assessed using the MELD score [20]. The MELD score is a mathematical formula that incorporates three biomarkers: serum bilirubin, serum creatinine, and the international normalized ratio (INR) of prothrombin time. It is a prognostic scoring system which predicts short-term survival in LC patients waiting for liver transplantation. Scores range from 6 to 40. To calculate the MELD score, blood samples were collected and analyzed for levels of the aforementioned biomarkers with standard automated laboratory methods.

### 2.6. Nutrition Screening

Two multidimensional tools, SGA and MNA, were used for the nutritional assessment [9,21]. The questionnaires were filled in and scores were calculated by the same trained dietitian.

The MNA questionnaire contains 18 items grouped into 4 categories: anthropometric measurements (BMI, weight loss, arm, and calf circumferences), general assessment (lifestyle, medication, mobility, and signs of depression or dementia), dietary assessment (number of meals, food and fluid intake), and self-perception of food and nutrition. Individual items have weighted scores. The total score ranges from 0 to 30. According to MNA scores, patients are classified as “well-nourished” (score ≥ 24), “at risk for malnutrition” (17–23.5), or “malnourished” (<17).

The SGA questionnaire includes patient’s history (changes in weight and dietary intake, gastrointestinal symptoms, and functional capacity) and a physical examination (subcutaneous fat, muscle wasting, and the presence of edema or ascites). Each variable is measured on a qualitative three-point scale. According to SGA rating, patients are classified as “well nourished” (A), “moderately nourished”/“suspected of being malnourished” (B), or “severely malnourished” (C).

### 2.7. Statistics

Descriptive statistics used for scale variables were the mean, median, and interquartile range (IQR), and absolute and relative frequency was used for nominal ones. The statistical significance among groups was tested with Pearson’s χ^2^ for nominal variables, and Mann–Whitney *U* or Kruskal–Wallis tests were used for scale variables of 2 or >2 independent groups, respectively. The covariance of ordinal or scale variables was tested with Spearman’s rho. The statistical significance of the effect of nominal parameters on Kaplan–Meier survival curves was tested with the log rank (Mantel–Cox) test. The performance of prognostic variables for survival was tested by receiver-operating characteristics (ROC) analysis and their prognostic accuracy was compared using the DeLong method. SPSS version 20 (IBM, Armonk, NY, USA) and Graphpad Prism (GraphPad Software, San Diego, CA, USA) were both used as software.

## 3. Results

Sixty-eight patients with compensated LC and sixty-nine with decompensated LC, recruited over 24 months, were followed up for similar median (IQR) periods of 19.3 (9) and 18 (18.5) months, respectively. The same demographic, anthropometric, nutritional, and clinical data recorded for patients were also collected from a control group of 148 healthy individuals. All three groups (controls, compensated and decompensated LC) were well balanced regarding sex, age, and LC etiology. Of note, non-alcoholic steatohepatitis (NASH) and alcohol etiologies were more abundant in decompensated than in compensated LC (26.1% vs. 16.2% and 29% vs. 13.2%, respectively; *p* < 0.01).

Anthropometrics, HGS, BIA, MNA, and SGA scores are presented in Table 1. BMI values did not differ among groups. Both patients and controls were classified as overweight or obese by BMI cut-off (≥25 kg/m^2^). Moreover, 60% and 43% of patients were of adequate nutritional status by SGA and MNA, respectively. Both MAC and TSF were lower in patients than controls (*p* < 0.023 and *p* < 0.017, respectively). The results for TSF remained statistically significant even after reducing statistical power by splitting cirrhotics into compensated and decompensated (*p* < 0.026). More patients with decompensated LC had low HGS adjusted for sex (29.4%) compared to patients with compensated LC (13.2%; *p* < 0.034) [5].

BIA provided a good number of useful parameters to assess emerging malnutrition, such as increased ECW (*p* < 10^−3^), decreased ICW (*p* < 10^−3^), BCM (*p* < 0.01), MM (*p* < 0.011), and Pha (*p* < 10^−3^). Deviations of most of the aforementioned parameters were more pronounced for decompensated cirrhotics (*p* < 0.048, *p* < 0.038, and *p* < 0.41 for ECW, ICW, and BCM, respectively; *p* < 0.01 for Pha). Setting age-adjusted thresholds on SMI (as measured by BIA) demonstrated that 21.3% of decompensated LC patients, 15% of compensated LC patients, and 11.5% of controls had reduced SMI. After splitting the cohorts to sexes, differences between cohorts were abrogated, and more males than females were classified as having reduced SMI in all cohorts of cirrhosis. In females, only 8% of decompensated LC patients had reduced SMI.

Both MNA and SGA scores correlated significantly with anthropometric measurements (Table 2). The scores also correlated with BIA-derived parameters, and the strongest were with the Pha. The MNA score was associated with HGS. The MELD score was strongly associated with SGA, but not with MNA.

**Figure 1:**
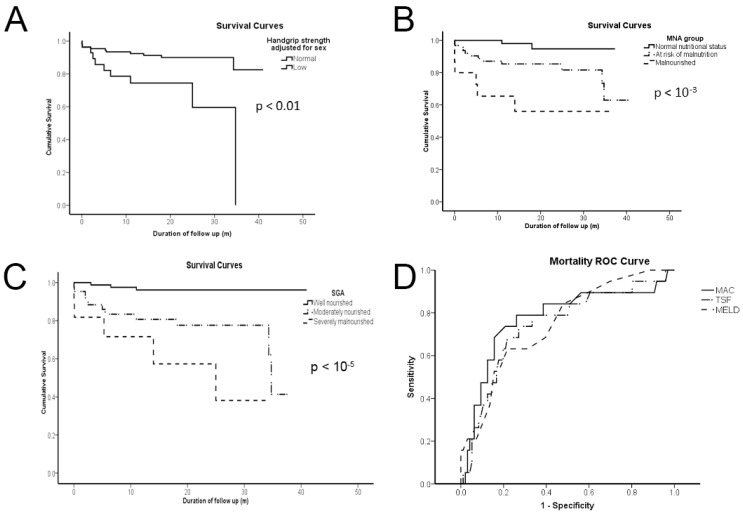
Low handgrip strength (**A**); malnutrition or risk of malnutrition by the mini-nutritional assessment (MNA) group (**B**); moderately or severely malnourished by the subjective global assessment (SGA) group (**C**); and triceps skinfold (TSF), mid-arm circumference (MAC), and the model for end-stage liver disease (MELD) score (**D**) are all of dismal predictive value for cirrhotics according to Kaplan–Meier survival curves of (**A**–**C**) and ROC curves (**D**). *p* values demonstrate statistical significance according to the log rank (Mantel–Cox) test for (**A**–**C**) and the DeLong method for (**D**).

During the follow-up period, 18 patients died (12 men and 6 women), of which 7 and 5 were malnourished, according to MNA and SGA, respectively, and 2 were well-nourished according to both scores. The mortality rate at follow-up was 13%. Patients who died belonged to the decompensated cohort. The causes of death were sepsis (*n* = 6), heart failure (*n* = 4), hepatorenal syndrome (*n* = 3), hepatic coma (*n* = 2), variceal hemorrhage (*n =* 2), and infectious respiratory disease (*n* = 1). Figure 1 illustrates the Kaplan–Meier survival curves of cirrhotics per HGS group adjusted for sex (A); the MNA group (B); the SGA group (C); and mortality ROC curves per TSF, MAC, and MELD score (D). Analyzing survival per HGS group adjusted for sex showed that cirrhotics with low HGS had a higher risk of mortality. Analysis per MNA or SGA group revealed that survival also differed per nutritional status. ROC curves of TSF, MAC, and MELD scores presented no statistically significant differences. SGA could not be compared since it is an ordinal variable; thus, it cannot even mimic a scale metric, as the MELD score. 

Beyond the MELD score, TSF and MAC of anthropometrics, HGS, most BIA parameters (TBW, ECW, ICW/ECW, FFM, BCM, FM, and Pha), and the MNA score could all predict mortality according to the asymptotic significance test (Table 3). Although TSF, MAC, Pha, and MNA had a higher AUC than the MELD score, differences did not reach statistical significance.

## 4. Discussion

The nutritional assessment of LC patients from Crete, Greece, demonstrated an unexpectedly high prevalence of well-nourished LC patients. However, patients had lower anthropometric and body composition indices than controls, and most anthropometric measurements fell between the 25th to 95th percentile of reference values for sex and age, reflecting an adequate nutritional status for the majority of patients. The finding was confirmed by the nutrition diagnostic questionnaires MNA and SGA, which identified 43.1% and 60.3% well-nourished patients, respectively. Based on the MNA and SGA scores, only 10.9% and 8.1% of the patients, respectively, were classified as being malnourished, whereas low HGS and SMI were found in 13.2% and 15% of compensated LC patients, and 29.4% and 21.3% of decompensated LC patients. Of note, malnutrition occurs in 20% of compensated patients and in more than 50% of decompensated patients, according to the literature [22].

Both patients and controls were classified as overweight or obese by BMI values, which could be attributed to a high prevalence of overweight and obesity in the general Greek population [23]. Although BMI and anthropometrics may be unreliable in LC patients because of fluid retention [24], MAC and TSF values were significantly lower in patients than controls in the present study, in agreement with previous studies [25]. This could be due to diuretic treatment and the fact that MAC and TSF are less affected by water retention than BMI [26]. The difference in TSF was maintained when patients were divided into compensated and decompensated, in line with results by Nunes et al. (2017) [27]. Furthermore, significantly more patients with decompensated LC had low HGS adjusted for sex, compared to patients with compensated LC, in accordance with Ciocîrlan and colleagues [6]. The combination of high BMI value (≥30 kg/m^2^), low TSF, muscle mass (MAMC and AMA), and HGS in malnourished patients is a sign of sarcopenic obesity, which is common in LC [28].

Deviations of BIA-derived Pha were more pronounced for decompensated cirrhotics. Pha values of decompensated cirrhotics were lower than those reported to be associated with mortality in LC [3]. Indeed, the patients who died were from the Pha ≤ 4.9° group. Female patients with reduced SMI belonged to the decompensated cohort, whereas male patients with reduced SMI were found in all cohorts of cirrhosis. These findings reflect sex-specific differences in LC and suggest that muscle depletion is more prevalent in male patients [29,30].

Generally, all nutritional assessment tools applied in the study revealed a relatively low prevalence of malnutrition in Cretan cirrhotic patients, even at the decompensated stage. This finding could be attributed to dietary habits which are more in line with the traditional Mediterranean diet. However, we did not include dietary interviews in order to assess dietary intakes or food consumption patterns, e.g., adherence to the traditional Mediterranean diet, which is a main limitation of this study.

Moreover, the study revealed several significant correlations. MNA and SGA scores were associated with most ΒΙA-derived parameters and anthropometric indices. HGS exhibited a significant association with MNA, in line with previous results in geriatric patients [31]. SGA was strongly associated with MELD scores, in agreement with results obtained by Ciocîrlan et al. [6]. This is the first study, to our knowledge, that compares MNA with SGA in LC patients, and both multidimensional tools proved to be significant in predicting malnutrition. The study also identified several predictors of mortality. Beyond the MELD score, TSF and MAC of anthropometrics, HGS, most BIA parameters (TBW, ECW, ICW/ECW, FFM, BCM, FM, and Pha), and MNA could all predict mortality. The MELD score has been shown to be a valid and independent predictor of short-term, as well as long-term, mortality in LC patients [32]. MAC, Pha, and MNA emerged as the best predictors of mortality with a predictive value at least equivalent to the MELD score. This study is the first to evaluate the prognostic value of MNA in this patient population, and its prognostic significance renders it an ideal tool for use on a regular basis to identify malnutrition in this patient population. Furthermore, the predictive value of MNA for mortality has also been demonstrated in oncological, end-stage renal disease, and heart failure patients [10,11,13].

The present study has several limitations that should be acknowledged. First, the nutritional assessment did not include any evaluation of dietary intakes or biochemical parameters, except for the biochemical markers for MELD score estimation. Moreover, physical activity was not assessed. Further research, including large-scale multicenter long-term follow-up studies, is required to confirm these findings. Nevertheless, this is the first study, to our knowledge, on the nutritional status of Greek liver cirrhosis patients. The strength of this study is the multiple screening tools used to assess malnutrition, which highlighted that the MNA score has equivalent predictive value for mortality to markers of disease severity (MELD scores) and objective measures of nutritional status, such as BIA-derived parameters. Additionally, the present study demonstrated that MAC, TSF, and HGS, which are simple and affordable bedside measurements, could detect nutritional deficits as effectively as sophisticated BIA. Since there is no single ideal tool to assess nutritional status in LC patients, a combination of various nutritional parameters is required [2]. Therefore, we propose routine evaluation of cirrhotics with a composite of MNA, MAC, TSF, and HGS to detect changes which can predict unfavorable events and evaluate therapeutic and nutritional interventions.

## Figures and Tables

**Table 1:** Characteristics of the patients and controls and their nutritional status. Numbers and percentages; mean values and standard deviations or 1st–3rd quartiles. Numbers represent N (% of group total) for nominal variables or median (IQR) for scale variables. Statistical significance was tested with Pearson’s χ^2^ for nominal variables. The Mann–Whitney *U* test was used for scale variables with 2 independent groups and the Kruskal–Wallis test was used for 3 independent groups. The *p* value on the left refers to comparisons between the control group and all cirrhotics. The *p* value in the middle refers to the comparison between the control group, compensated cirrhotics, and decompensated cirrhotics. The *p* value on the right refers to the comparison between compensated and decompensated cirrhotics.

	Controls	Cirrhosis:	Compensated	Decompensated	*p* <
**N**	148 (51.9)	137 (48.1)	68 (23.9)	69 (24.2)	
Demographics					
Female sex	68 (45.9)	61 (44.5)	32 (47.2)	29 (45.1)	0.810/0.816/0.554
Age (y)	65 (16)	67 (15)	69 (15)	66 (14)	0.152/0.280/0.461
Cause of cirrhosis					
NASH		29 (21.2)	11 (16.2)	18 (26.1)	0.137/0.01/0.01
Alcohol		29 (21.2)	9 (13.2)	20 (29)	
HCV		29 (21.2)	22 (32.4)	7 (10.1)	
HBV		25 (18.2)	12 (17.6)	13 (18.8)	
Autoimmune LD		25 (18.2)	14 (20.6)	11 (15.9)	
Follow up time (m)		19 (12.5)	19.3 (9)	18 (18.5)	-/-/0.188
**Deaths**	0 (0)	19 (13.9)	0 (0)	19 (27.5)	0.001/0.001/0.001
Anthropometrics					
BMI (kg/m^2^)	29 (6)	29.5 (6.5)	29.1 (6.4)	30.1 (6.5)	0.240/0.337/0.372
TSF (cm)	2.93 (1.73)	2.6 (1.67)	2.67 (1.43)	2.48 (1.77)	0.017/0.026/0.198
MAC (cm)	32 (6)	31.4 (5.3)	32 (3.8)	30.5 (6.1)	0.023/0.025/0.235
MAΜC (cm)	22.5 (5.2)	22.7 (4)	22.9 (3.7)	22.2 (4.3)	0.979/0.573/0.044
AMA (cm^2^)	40.6 (18.6)	40.7 (14.9)	41.6 (13.8)	39 (14/5)	0.773/0.422/0.156
HGS (kg)					
Low	24 (19.5)	28 (20.6)	9 (13.2)	20 (29.4)	0.829/0.100/0.034
(cut-off adjusted to sex)					
BIA					
Low SMI (%)	15 (11.5)	22 (18.2)	9 (15)	13 (21.3)	0.099/0.043/
Males	15 (19.7)	20 (29.4)	9 (28.1)	11 (30.6)	0.112/0.080/
Females	0 (0)	2 (3.8)	0 (0)	2 (8)	0.146/0.034/
TBW (%)	51.8 (10.1)	51.5 (6.7)	51.2 (10.3)	51.5 (8.6)	0.662/0.805/0.611
ECW (%)	46.7 (6)	48.2 (7.5)	47.8 (7.8)	49.1 (9.3)	10^−3^/10^−3^/0.048
ICW (%)	53.3 (6)	51.9 (7.6)	52.3 (7.9)	50.9 (9.3)	10^−3^/10^−3^/0.038
ICW/ECW (%)	1.1 (0.2)	1.1 (0.3)	1.1 (0.3)	1 (0.4)	10^−3^/10^−3^/0.030
FFM (%)	67.5 (13.9)	65.6 (14.3)	64.6 (14.3)	66.4 (12.4)	0.349/0.300/0.217
BCM (%)	48.4 (11.9)	45 (15.4)	46.1 (12.8)	42.8 (13.4)	0.01/0.01/0.041
FM (%)	32.8 (14.3)	34.4 (14.6)	35.4 (14.9)	33.6 (12.4)	0.429/0.328/0.208
Pha (^o^)	5.5 (1.6)	5 (1.5)	5.3 (1.5)	4.8 (1.4)	10^−3^/10^−4^/0.01
Nutritional assessment					
MNA score	25.5 (5.5)	22.5 (3.7)	24.3 (5.5)	22 (5)	10^−6^/10^−6^/0.026
Normal	104 (70.3)	58 (43.1)	37 (54.4)	31.9	
At risk	42 (28.4)	64 (46)	26 (38.2)	53.6	
Malnourished	2 (1.4)	15 (10.9)	5 (7.4)	14.5	
SGA					10^−8^/10^−15^/10^−6^
A	135 (91.2)	82 (60.3)	56 (82.4)	26 (38.2)	
B	12 (8.1)	43 (31.6)	11 (16.2)	32 (47.1)	
C	1 (0.7)	11 (8.1)	1 (1.5)	10 (14.7)	

HBV: hepatitis B virus; HCV: hepatitis C virus; LD: liver disease; NASH: non-alcoholic steatohepatitis; PLT: platelets; BMI: body mass index; AMA: arm muscle area; MAC: mid-arm circumference; MAMC: mid-arm muscle circumference; TSF: triceps skinfold; HGS: handgrip strength; BIA: bioimpedance analysis; SMI: skeletal muscle index; BCM: body cell mass; TBW: total body water; ECW: extracellular water; ICW: intracellular water; FFM: fat-free mass; FM: fat mass; Pha: phase angle; MNA: mini nutritional assessment; SGA: subjective global assessment.

**Table 2:** Correlations of nutritional scores with several parameters. Spearman’s rho value and its statistical significance are also reported.

Nutritional Scores	Anthropometrics	Dynamometry	BIA	Composite Scores
MNA	MAMC (0.246, 0.01)	HGS (0.265, 0.01)	Pha (0.482, 10^−7^)	
	MAC (0.24, 0.01)		ICW (0.384, 10^−4^)	
	AMA (0.237, 0.01)		ECW (−0.383, 10^−4^)	
	BMI (−0.229, 0.014)		ICW/ECW (0.383, 10^−4^)	
			SMI (0.293, 0.01)	
			BCM (0.226, 0.039)	
SGA MNA (−0.595, 10^−13^)	MAC (−0.277, 0.01)		Pha (−0.536, 10^−9^)	MELD (0.43, 10^−5^)
	TSF (−0.202, 0.018)		ICW/ECW (−0.428, 10^−5^)	
			ECW (0.421, 10^−5^)	
			ICW (−0.401, 10^−5^)	
			BCM (−0.325, 10^−3^)	
			SMI (−0.276, 0.01)	

AMA: arm muscle area; BIA: bioimpedance analysis; BCM: body cell mass; BMI: body mass index; ECW: extracellular water; FFM: fat-free mass; FM: fat mass; HGS: handgrip strength; ICW: intracellular water; MELD: model for end-stage liver disease score; MAC: mid-arm circumference; MAMC: mid-arm muscle circumference; Pha: phase angle; SMI: skeletal muscle index; TSF: triceps skinfold.

**Table 3:** Receiver operating characteristic (ROC) curves for mortality from cirrhosis. Area under the curve (AUC), 95% confidence interval (CI), and statistical significance (*p*) versus the useless test are provided.

Factor	AUC	95% CI	*p* <
MELD score	0.747	0.632–0.862	10^−3^
Anthropometrics			
BMI (kg/m^2^)	0.592	0.44–0.744	0.189
TSF (cm)	0.755	0.632–0.878	10^−3^
MAC (cm)	0.787	0.66–0.915	10^−4^
MAMC (cm)	0.585	0.756–0.714	0.226
AMA (cm^2^)	0.584	0.456–0.711	0.232
HGS (kg)	0.66	0.543–0.777	0.022
BIA			
SMI (kg/m^2^)	0.505	0.349–0.66	0.948
TBW (%)	0.727	0.594–0.861	0.01
ECW (%)	0.709	0.547–0.871	0.01
ICW (%)	0.708	0.545–0.871	0.01
ICW/ECW (%)	0.701	0.54–0.863	0.01
FFM (%)	0.689	0.542–0.836	0.013
BCM (%)	0.684	0.533–0.834	0.016
FM (%)	0.689	0.542–0.836	0.013
Pha (^o^)	0.765	0.633–0.897	10^−3^
Nutritional assessment			
MNA	0.765	0.657–0.874	10^−3^

AMA: arm muscle area; BCM: body cell mass; BIA: bioimpedance analysis; BMI: body mass index; ECW: extracellular water; FFM: fat-free mass; FM: fat mass; HGS: handgrip strength; ICW: intracellular water; MAC: mid-arm circumference; MAMC: mid-arm muscle circumference; MNA: mini nutritional assessment; Pha: phase angle; SMI: skeletal muscle index; TBW: total body water; TSF: triceps skinfold.

## Data Availability

The data presented in this study are available on request from the corresponding author. The data are not publicly available, as this is not in accordance with consent provided by participants on the use of confidential data.
